# More than meets the (human) eye: Cryptic chromatic diversity in a colour polymorphic lizard: Cryptic polymorphism in lacertid lizards

**DOI:** 10.1007/s00114-026-02106-2

**Published:** 2026-05-07

**Authors:** Guillem Pérez i de Lanuza, Enrique Font

**Affiliations:** Ethology Lab, Cavanilles Institute of Biodiversity and Evolutionary Biology, University of Valencia, APDO 22085, 46071 València, Spain

**Keywords:** Colour polymorphism, Cryptic polymorphism, *Podarcis*, Ultraviolet, Visual modelling

## Abstract

Understanding the evolution of polymorphisms, such as colour polymorphisms, is a key aim in evolutionary biology. Accurate, objective description of the different colour variants is crucial to correctly explore colour polymorphisms, a task that can be hindered by the existence of cryptic morphs. Colour polymorphisms are common in many lizards, including lacertids (fam. Lacertidae). The common wall lizard, *Podarcis muralis*, shows a complex colour polymorphism involving up to five discrete morphs that differ in their ventral coloration: white, yellow, and orange pure morphs, as well as white-orange and yellow-orange mosaic morphs. Here we report that colour polymorphism in this species is more complex than hitherto appreciated due to the presence of two different types of white coloration: one absorbing and another reflecting in the near ultraviolet (UV). Our results show that both colours are found in all populations although in different frequencies. Visual models indicate that both whites are likely to be perceived by the lizards as distinct colour categories. No equivalent cryptic colour variation was found among yellow or orange animals. However, the two whites are also present in white-orange mosaics. As both whites are present in different adult individuals from the same population, it seems more adequate to describe the ventral polymorphism of *P. muralis* as comprising not five, but seven morphs: UV-absorbing white (UV^−^white), UV-reflecting white (UV^+^white), yellow and orange pure morphs, as well as UV^−^white-orange, UV^+^white-orange, and yellow-orange mosaic morphs. Our results demonstrate that the two types of white are also found in other *Podarcis* species as well as in other lacertids, revealing that this phenomenon is not a derived character restricted to *Podarcis*. The existence of cryptic morphs highlights the impact of relying on anthropomorphic descriptions in studies of colour polymorphisms.

**Supplementary Information:**

The online version contains supplementary material available at 10.1007/s00114-026-02106-2.

## Introduction

Polymorphisms, meaning the coexistence of two or more different genetically-determined phenotypes within the same interbreeding population (Ford 1945; Gray and McKinnon 2007), provide unique and fascinating case studies to investigate complex evolutionary processes. Colour polymorphic species in particular have attracted much interest from evolutionary biologists. Polymorphisms involving colour variants are often conspicuous and easily discriminable by the human visual system. Colour morphs may differ in traits other than their chromatic properties, often representing alternative phenotypic optima resulting from complex evolutionary processes (Gray and McKinnon 2007; McKinnon and Pierotti 2010; Svensson 2017; Wellenreuther et al. 2014; White and Kemp 2016). The study of colour polymorphisms has served to test many hypotheses about the maintenance of biological diversity (Svensson 2017). Studies describing the coexistence of different morphs due to their association with alternative reproductive tactics (Lank et al. 1995; Sinervo and Lively 1996), or sympatric speciation via sensory drive mechanisms combined with assortative mating (e.g. Maan and Sefc 2013) are now considered paradigmatic. However, identifying and describing colour polymorphisms is not always easy and has given rise to several controversies regarding whether colour variation shown by organisms represents a polymorphism, and the number of morphs that it encompasses (e.g. Vercken et al. 2008; Cote et al. 2008; Paterson and Blouin-Demers 2017).

Colour variation can be discrete (involving several categorical morphotypes) or continuous (representing a gradual variation in colour; Roulin 2004). Knowing the way in which this variation is perceived by relevant receivers (i.e. conspecifics, predators) is crucial to correctly identify the evolutionary units of interest for understanding the causes and consequences of the polymorphism (Bennett et al. 1994; Eaton 2005). Descriptions that do not consider the point of view of observers, such as those based solely on our own (i.e. human) visual perception, could be insufficient to conclude that natural chromatic variation is an actual polymorphism (Teasdale et al. 2013; Pérez i de Lanuza et al. 2018a). Ignoring how relevant observers perceive colours could erroneously inflate the number of colour morphs beyond what conspecifics can actually discriminate, but also lead to oversight of potential cryptic colour variation. For example, in the sailfin silverside *Telmatherina antoniae*, initially males were considered polymorphic and females monomorphic. However, an objective study of their coloration using reflectance spectrophotometry demonstrated that females are also polymorphic (Pfaender et al. 2014).

Colour is crucial in many aspects of animal biology, especially in reptiles, where it is involved in communication, camouflage, aposematism, mimicry, thermoregulation, and species recognition (Olsson et al. 2013; Caro et al. 2017). Colour polymorphisms are common in lizards, involving diverse clades such as phrynosomatids, agamids, and sphaerodactylids, which show a strong convergence in morph colours (Stuart-Fox et al. 2020). They are particularly prevalent in the family Lacertidae (Stuart-Fox et al. 2020; Brock et al. 2022; de Solan et al. 2023) and research conducted with this group has led to relevant advances in our understanding of the evolution of colour polymorphism. For instance, studies with *Zootoca vivipara* provided the first experimental evidence supporting the idea that colour polymorphisms can be maintained by negative frequency-dependent selection in what is known as a rock-paper-scissors evolutionary game (Fitze et al. 2014; San-José et al. 2014).

Wall lizards (genus *Podarcis*) have complex colour polymorphisms involving white, yellow, and orange/red ventral colours, and have attracted much attention (e.g. *P. melisellensis*, Huyghe et al. 2007; P. *muralis*, Calsbeek et al. 2010; P. *gaigeae*, Runemark et al. 2010; P. *erhardi*, Brock et al. 2020). Several recent studies have examined the colour polymorphism of *Podarcis muralis* (Laurenti, 1768), a species with a wide native distribution range spanning much of mainland Europe (Speybroeck et al. 2016) and introduced populations in southern Britain and North America (e.g. Michaelides et al. 2015). The ventral polymorphism shown by this species comprises five discrete morphs: three pure morphs − white, yellow, and orange − and two mosaics − white-orange and yellow-orange (Cheylan 1988; see details in Pérez i de Lanuza et al. 2013a). These colour morphs are present in both males and females, fixed before or around the time the lizard reaches sexual maturity, and genetically determined (Pérez i de Lanuza et al. 2013a; Andrade et al. 2019). Two independently segregating autosomal genes related to the metabolism of pterins and carotenoids explain the presence of yellow and orange pigments, and epistatic interactions between them modulate intramorph variation (Andrade et al. 2019; Aguilar et al. 2022). Reflectance spectrophotometry reveals that the chromatic differences between ventral morphs in *P. muralis* are accounted by the overall shape of the spectral curve (reflecting differences in luminance and chroma), rather than by the location of their reflectance peak (hue), which is similar in all morphs and therefore less informative (see Fig. 2 in Pérez i de Lanuza and Font 2015). Colour discrimination experiments and visual modelling confirm that *P. muralis* can discriminate the three colours (white, yellow, and orange) involved in the polymorphism, this being one of the few species for which there is experimental evidence showing that the lizards perceive their own ventral colour variation as discrete colour categories (Pérez i de Lanuza et al. 2018a).

Although the use of visual models to determine the discriminability of colour morphs is now commonplace in studies with polymorphic lizards, initial colour categorization is still largely based on human visual assessment. This is problematic because it can lead to underestimating chromatic variation and therefore the number of colour morphs (Hews and Martins 2013; Stuart-Fox et al. 2003; Ossip-Drahos et al. 2016). The white colour morph in *Podarcis muralis* is a case in point. Surfaces that reflect all wavelengths equally are generally perceived as white under natural viewing conditions (Bosten et al. 2015). Thus, we perceive the colour of the white *P. muralis* morph as white because it reflects across the entire human visual spectrum (400–700 nm). However, as lizards have cone photoreceptors with peak sensitivities in the near ultraviolet spectrum (UV; i.e. 320–400 nm, Pérez i de Lanuza and Font 2014; Martin et al. 2015), they cannot perceive as white a colour that reflects between 400 and 700 nm but absorbs in the near UV range (see Fig. 2 in Pérez i de Lanuza and Font 2015). True “lizard white” must show reflectance across the entire sensitivity range of lizards (i.e. 320–700 nm).

In many populations of *P. muralis* from the Pyrenees, the yellow and orange coloration of females does not extend over the entire ventral surface and is restricted to the gular area, the belly remaining white. Although not explicitly recognized, reflectance spectra from our own previous studies show that some white females display two different types of white coloration in their throats and bellies (see Fig. 2 in Pérez i de Lanuza and Font 2015): UV-absorbing white (UV^−^white) that reflects in the 400–700 nm range, and UV-reflecting white (UV^+^white) that reflects across the 300–700 nm range. Only the latter corresponds to true “lizard white”. Similarly, juveniles are UV reflecting (UV^+^white) across their entire ventral surface, losing this coloration during development (Ábalos et al. 2025). Two types of white coloration have also been documented in birds, where white feathers that either reflect or do not reflect UV have been described (Burkhardt 1996; Burkhardt and Finger 1991). However, the implications of these two types of white for assessing colour variation in polymorphic lizards remain unexplored. In particular, the occurrence of two distinct whites (UV^+^white and UV^−^white) raises the possibility that colour polymorphism in *P. muralis*, and potentially in other lizard taxa, includes cryptic morphs that have so far escaped detection.

Here we examine ventral colour variation in *P. muralis* to determine if this species shows cryptic colour polymorphism involving UV^+^white and UV^−^white coloration. Previous studies with Pyrenean *P. muralis* have shown strong geographic variation in local morph composition, probably driven by differences in the intensity of sexual selection (Pérez i de Lanuza et al. 2013a, 2017; Aguilar et al. 2024) and by environmental factors such as humidity (Pérez i de Lanuza and Carretero 2018; Pérez i de Lanuza et al. 2018b). Therefore, our study encompasses 17 Pyrenean populations differing in morph diversity to evaluate if local frequencies of the potential cryptic morphs also vary geographically. Finally, as ventral white coloration is widespread in the genus *Podarcis* and other lacertids, we also explore available spectra of other species to determine if this type of chromatic variation also occurs in other species. For clarity, we use the term UV^−^white for colour patches reflecting between 400 and 700 nm (i.e. UV-absorbing white), UV^+^white for colour patches reflecting between 320 and 700 nm (i.e. UV-reflecting white), and white as a generic term to include any colour that we perceive as white.

## Materials and methods

### Study species

**Fig. 1:**
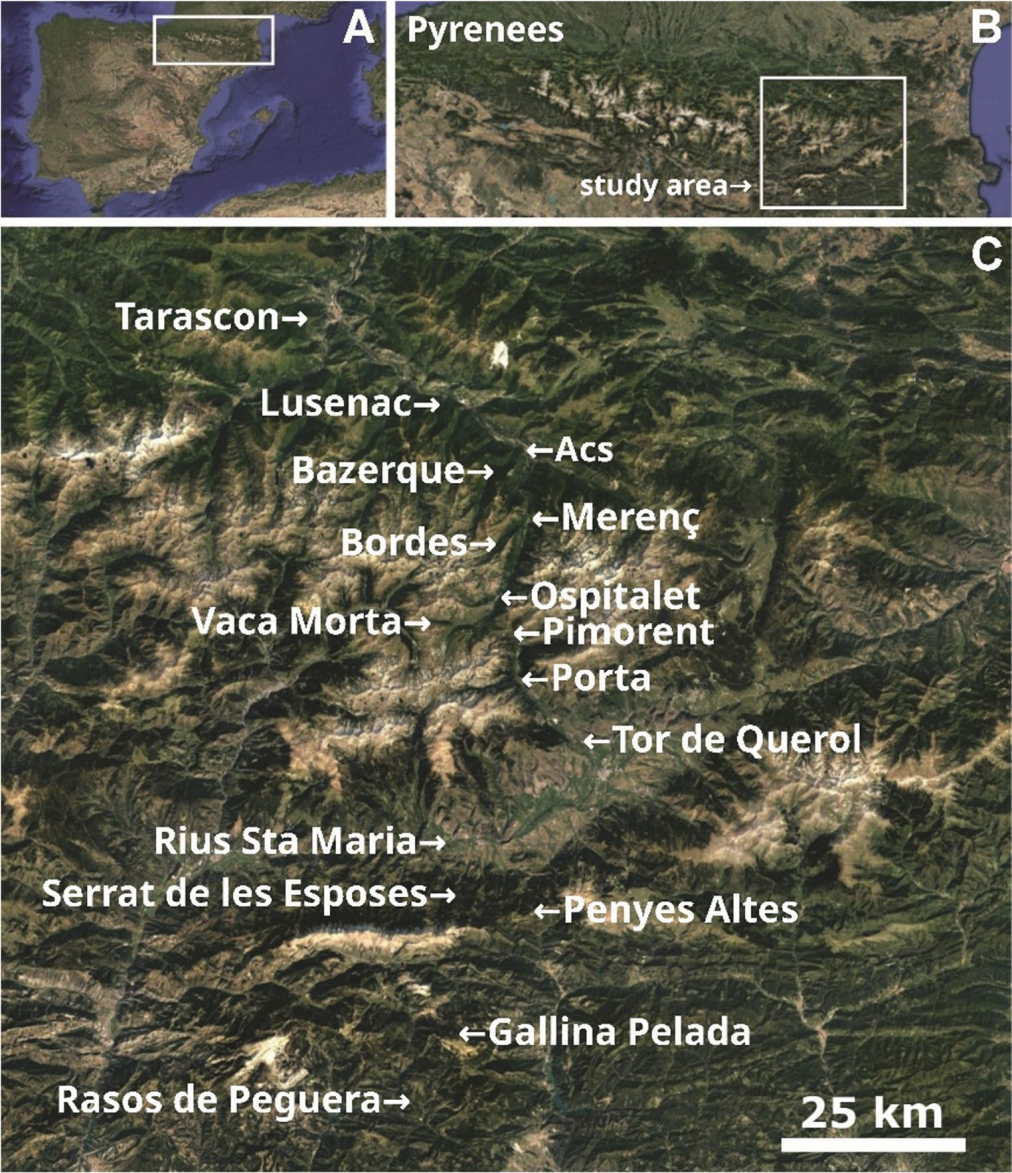
Sampling area of *Podarcis muralis* in the Pyrenees. (**A**) General view of the Iberian Peninsula. (**B**) Enlarged view of the Pyrenees showing the location of our study area (white square). (**C**) Detail of the study area, showing the sampling localities. Maps data: Google 2025

During the breeding season (i.e. spring-summer) of 2018–2020 we sampled 1068 adult *P. muralis* in 17 Pyrenean localities (Fig. 1; Table S1). From this sample, 614 lizards belonging to the white or white-orange (i.e. mosaic) morphs were used to determine the prevalence and spectral characteristics of UV^+^white and UV^−^white ventral coloration using objective techniques of colour measurement and considering the visual system of the species (i.e. spectral analyses and visual modelling, respectively).

**Fig. 2:**
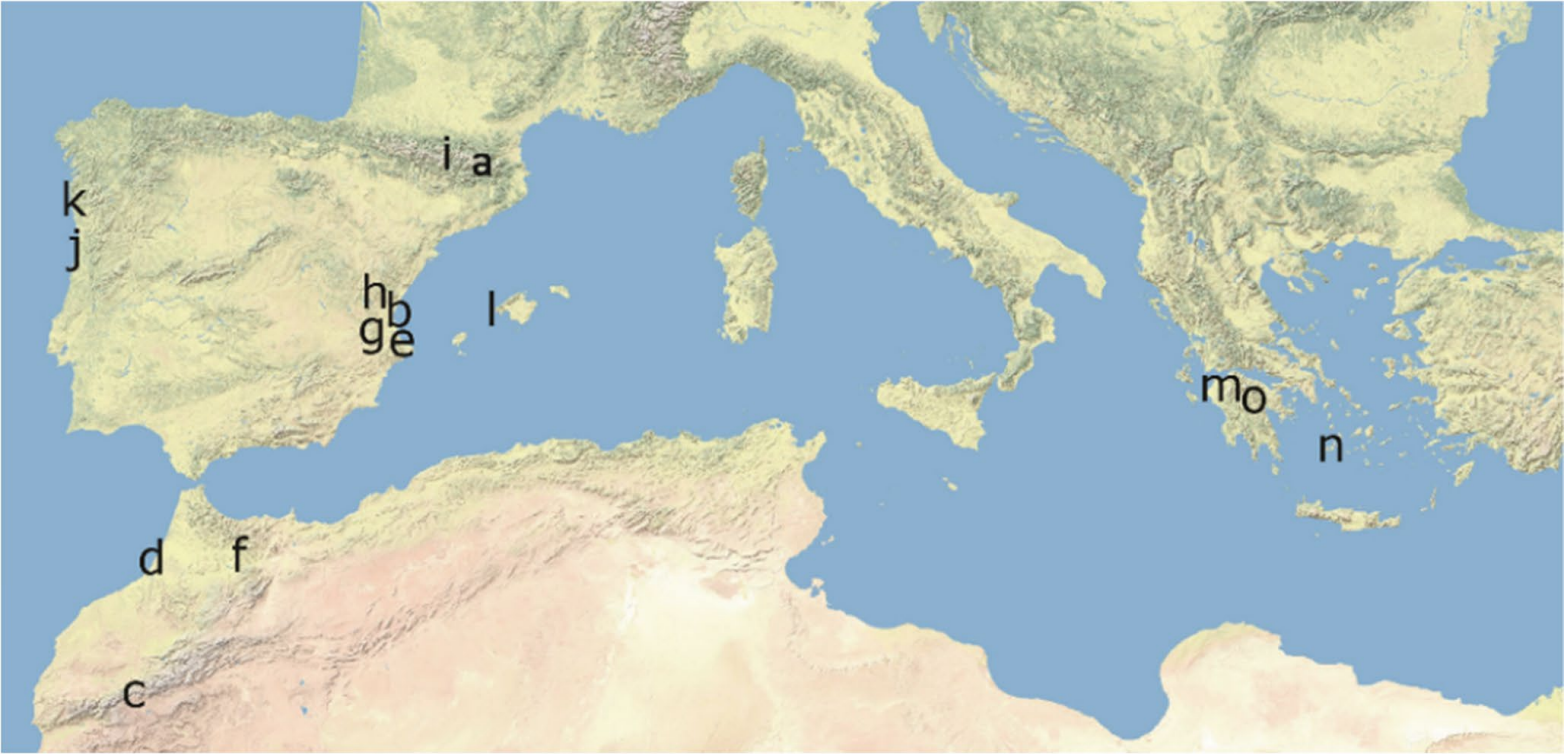
Sampling localities: a = sampling area for *Podarcis muralis* (see Fig. 1); b = *Psammodromus edwarsianus* and *Acanthodactylus erythrurus*; c = *Atlantolacerta andreanskyi* and *Podarcis vaucheri*; d = *Acanthodactylus lineomaculatus*; e = *Timon nevadensis*; f = *Scelarcis perspicillata*; g, h and i = *Podarcis liolepis*; j = *P. carbonelli*; k = *P. lusitanicus*; l = *P. lilfordi gigliolii*; m = *P. ionicus* and *P. peloponnesiacus*; n = *P. milensis*; o = *P. thais*

To assess the prevalence of the UV^+^white and UV^−^white coloration in other lacertid species, we examined spectra of ventrally white adult lizards belonging to other species available in our cumulative database of lizard spectra. In particular, we used spectra from one population of *Podarcis lusitanicus* (Moledo), one of *P. carbonelli* (Torreira), three populations of *P. liolepis* (Godella, Alzira, Espot), one of *P. lilfordi* (Dragonera island, Mallorca), and one population each of *P. vaucheri* (Oukaimeden), *P. peloponnesiacus* (Feneos), *P. ionicus* (Feneos), *P. thais* (Stymphalia) and *P. milensis* (Milos). In addition, we examined spectra from species belonging to other lacertid genera: *Psammodromus edwarsianus*, *Acanthodactylus erythrurus*, *A. lineomaculatus*, *Atlantolacerta andreanskyi*, *Timon nevadensis*, and *Scelarcis perspicillata pellegrini*. Spectra from many of these species had been collected and used in previous studies for purposes other than studying polymorphism (Font et al. 2009; Pérez i de Lanuza et al. 2013b; de la Cruz et al. 2023). Sampling locations for these species are shown in Fig. 2 and sample sizes can be found in Table S2. The choice of these species is opportunistic, but encompasses representatives from all the major groups within the family Lacertidae.

### Reflectance spectrophotometry

Reflectance spectra from lizard throats and bellies were collected with a USB-2000 portable spectrometer and a PX-2 xenon light source (both from Ocean Optics Inc.; Dunedin, USA), calibrated with a Spectralon white diffuse reflectance standard (Labsphere) (see Badiane et al. 2017 for technical details). We excluded individuals showing a pre-moulting aspect to avoid any spectral distortions. Measurements of white-orange mosaics, in both the throat and the belly, were obtained only when the size of the white patches was large enough (ca. > 2 mm in diameter) to allow proper measurements, thus avoiding chimeric spectra (Badiane et al. 2017).

Spectra from throats and bellies were classified as UV^+^white or UV^−^white based on the wavelength at which reflectance is halfway between its minimum and its maximum values (R_mid_; Pryke et al. 2001; Smiseth et al. 2001) in the 300–450 nm wave-range. Spectra were classified as UV^+^white when R_mid_ values were equal or below 345 nm, and as UV^−^white when R_mid_ values were equal or above 365 nm (Ábalos et al. 2025).

After spectra classification, luminance (i.e. the sum of the reflectance across the 300–700 range), ultraviolet chroma (C_UV_; i.e. the sum of the reflectance across the 300–400 range divided by the sum of the entire range), and peak location (which represents a measure of hue, complementary to R_mid_) were extracted from spectra using the package *pavo 2* (Maia et al. 2019) in R 4.4.2 (R Development Core Team, 2024). After graphically exploring the residuals, we assumed a Gaussian distribution and used LMM to test if the spectral variables (i.e. luminance, C_UV_, and hue) differ between the UV^+^white and UV^−^white categories with morph and sex as fixed factors, the interaction between both factors, SVL as continuous predictor, and population as a random factor. We repeated these analyses for white throats, white bellies, white-orange throats, and white-orange bellies using the *lme4* package.

### Visual modelling

We used visual models to determine the chromatic distances between the *P. muralis* spectra classified as UV^−^white and UV^+^white. We performed these analyses using Vorobyev and Osorio’s (1998) receptor noise model, with cone sensitivities of *P. muralis* (Martin et al. 2015). Chromatic distances were calculated assuming a cone abundance ratio of 1:1:1:4 (corresponding to the ultraviolet-, short-, middle- and long-wavelength-sensitive cones; Martin et al. 2015), and a Weber fraction of 0.05 for the long-wavelength sensitive cone (Marshall and Stevens 2014; Martin et al. 2015; Pérez i de Lanuza et al. 2018a). Chromatic and achromatic distances are expressed as just noticeable differences (JND). Values above 3 JND are easily discriminable, and chromatic distances between 1 and 3 JND are discriminable only under good illumination conditions (Siddiqi et al. 2004). Visual models were constructed using *pavo 2* (Maia et al. 2019). We used the 2-step analysis proposed by Maia and White (2018) to estimate colour discrimination statistically and perceptually between the UV^−^white and UV^+^white categories. Thus, we used the *bootcoldist* function of *pavo 2* to obtain geometric mean chromatic distances between both white categories and to estimate 95% confidence intervals through a bootstrap procedure, and a distance-based PERMANOVA using the *adonis2* function from the R package *vegan* 2.6-4 (Oksanen et al. 2022).

## Results

**Fig. 3:**
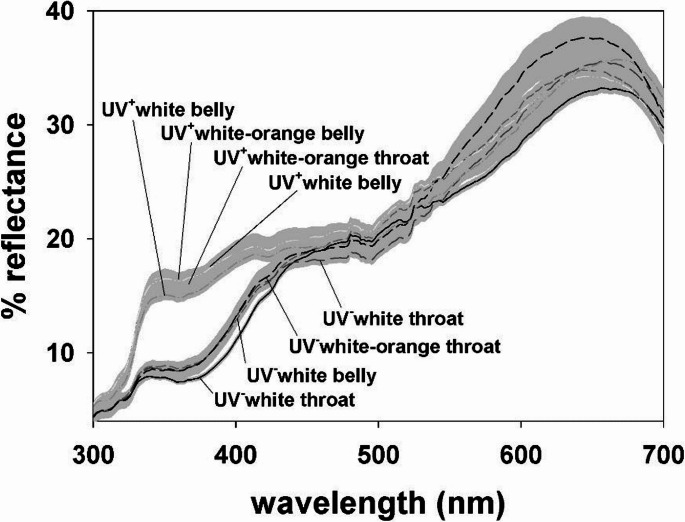
Reflectance spectra of pure UV^−^white and UV^+^white, and of white patches of UV^−^white-orange and UV^+^white-orange mosaics. Throat and belly spectra are depicted separately. Lines represent mean spectra and grey areas represent the associated standard error of the mean. Sample sizes are given in Table 1

**Fig. 4:**
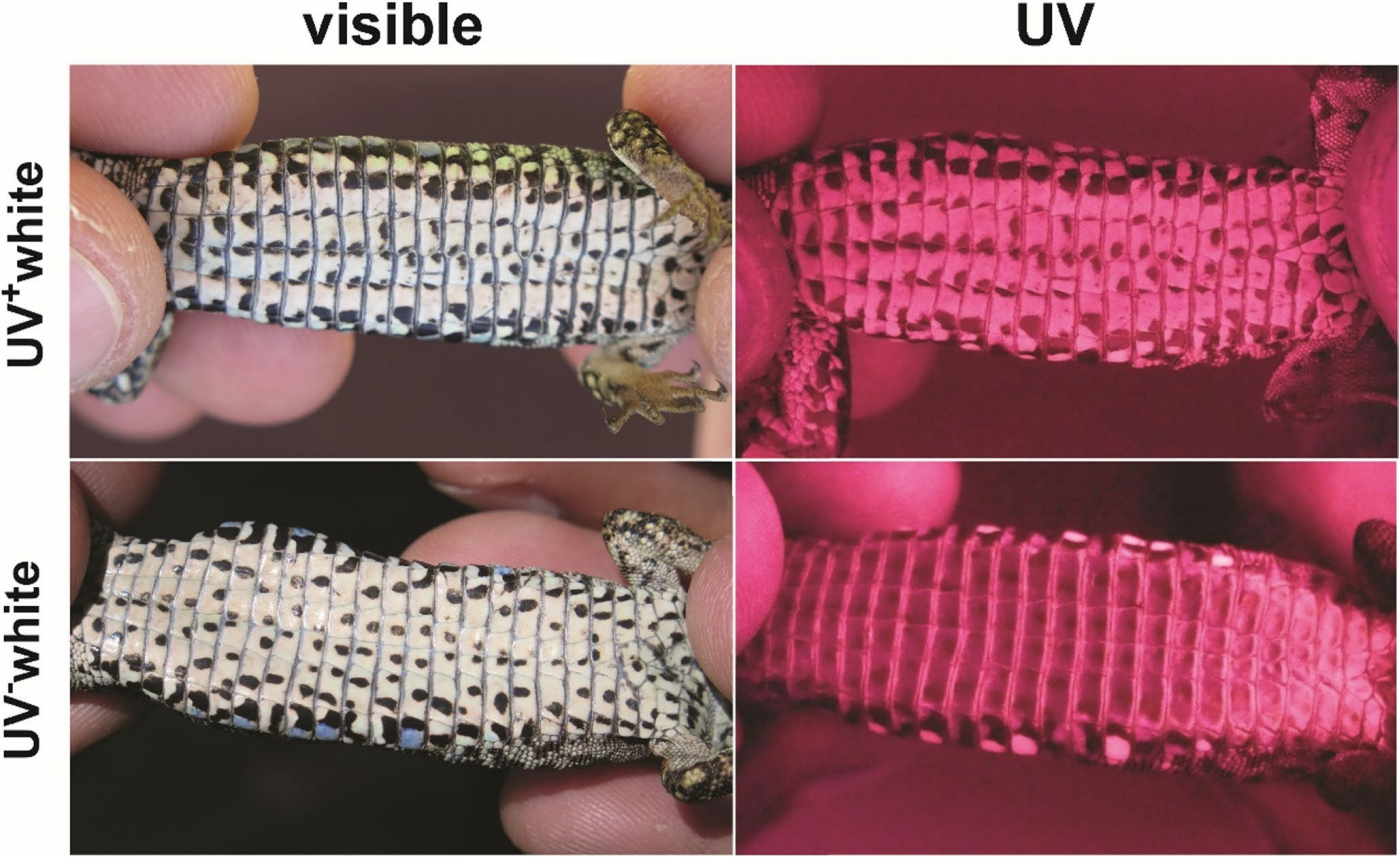
Representative pictures of male *Podarcis muralis* showing the UV^+^white phenotype (top) and the UV^−^white phenotype (bottom), in the visible spectrum (i.e. 400–700 nm; left) and in the near UV spectrum (i.e. between 320–380 nm; right). The lizards’ head is to the right in all the pictures. UV-reflecting skin patches in the UV pictures are visible due to their lighter, whitish colouration. The UV^−^white male’s throat and belly look dark in the UV picture because they absorb wavelengths below 400 nm. In contrast, the ventral surface of the UV^+^white male looks much lighter in the UV picture because it reflects in the near UV. Both phenotypes show a homogenous pattern across the entire ventral surface. Pictures in the human-visible range were obtained with a standard digital camera (Canon PowerShot G16). UV pictures were obtained with a digital camera (Olympus PEN Mini) with the standard internal hot mirror filter replaced by a Spectrosil 2000 fused silica filter, and a UV-transmitting macro lens (Noflexar Novoflex 1:3,5/35 mm) and a Baader U-filter with peak transmission at 350 nm (ca. 80%) and a bandwidth of 60 nm (between 320 and 380 nm). UV photographs were taken outdoors in the shade using natural illumination. Note that the blue patches located along the lizards’ flanks are highly UV reflective

No orange or yellow-orange (i.e. mosaic) *P. muralis* lizards show UV reflectance ventrally. Yellow animals likewise did not show UV reflectance, except for a single individual with a secondary reflectance peak in the near UV wavelength range (1% of yellow individuals in our sample; Figure S2). In contrast, white and white-orange lizards show two distinct types of ventral white coloration differing in spectral properties. One type reflects in the near UV (UV^+^white), and the other absorbs in this part of the spectrum (UV^−^white). Results are similar for throats and bellies, as well as for the white patches present in throats and bellies of white-orange mosaics (Fig. 3). The two whites differ in luminance and especially in ultraviolet chroma (C_UV_), sex and body size playing a secondary role in this colour variation (Table 1; Fig. S1). Most white males have a uniform ventral pattern, with both throat and belly either UV^+^white or UV^−^white (Table 2). White females often have a UV^+^white belly regardless of the white of their throat, which can be either UV^+^white or UV^−^white. However, some females show UV^−^white throats and bellies (Table 2). Figure 4 shows pictures in the human-visible and UV range of two representative males, one with UV^+^white ventral coloration and the other with UV^−^white coloration.

**Table 1:** Results of LMM analyses. Morph and sex were considered as fixed factors, SVL as continuous predictor, and population as a random factor. Sample sizes classified by white type and sex (m=males, f=females) in parenthesis. Analyses were independent for white throats, white bellies, white-orange throats, and white-orange bellies

		white throat (UV^−^white-m = 110; UV^−^white-f = 135; UV^+^white-m = 115; UV^+^white-f = 80)					white-orange throat (UV^−^white-m = 32; UV^−^white-f = 75; UV^+^white-m = 18; UV^+^white-f = 49)				
		Estimate	SE	df	t	*P*	Estimate	SE	df	t	*P*
luminance	morph	1060	186	426.4	5.69	< 0.001	785.7	260.9	165.7	3.01	0.003
	sex	-646	166	424.5	-3.89	< 0.001	-693.4	291.0	161.1	-2.38	0.018
	SVL	-29	15	434.3	-1.94	0.053	-14.7	24.9	161.0	-0.59	0.555
	morph*sex	145	257	429.0	0.56	0.573	-345.8	471.0	160.8	-0.73	0.464
C_UV_	morph	-0.020	0.003	431.0	-7.61	< 0.001	0.038	0.004	168.9	9.96	< 0.001
	sex	0.006	0.002	427.6	2.54	0.012	0.005	0.004	166.0	1.18	0.241
	SVL	0.001	0.000	434.4	2.65	0.008	0.001	0.000	165.2	-1.50	0.135
	morph*sex	-0.001	0.004	433.9	-2.36	0.019	0.001	0.006	165.0	0.02	0.986
hue	morph	4.75	2.49	427.2	1.91	0.057	5.156	3.32	168.1	1.55	0.122
	sex	-9.91	2.22	424.5	-4.47	< 0.001	-10.425	3.72	163.8	-2.81	0.006
	SVL	0.44	0.20	435.7	2.19	0.029	0.922	0.32	163.2	2.90	0.004
	morph*sex	0.77	3.43	430.3	0.23	0.822	0.669	6.02	163.0	0.11	0.912
		white belly (UV^−^white-m = 134; UV^−^white-f = 135; UV^+^white-m = 105; UV^+^white-f = 298)					white-orange belly (UV^−^white-m = 12; UV^−^white-f = 2; UV^+^white-m = 5; UV^+^white-f = 16)				
		*Estimate*	*SE*	*df*	*t*		*Estimate*	*SE*	*df*	*t*	
luminance	morph	366.5	159.2	658.6	2.30	0.022	-372.5	1402.3	28.6	-0.27	0.792
	sex	-766.2	180.8	654.4	-4.24	< 0.001	-1413.3	1479.1	29.2	-0.96	0.347
	SVL	-27.5	13.9	661.4	-1.97	0.049	-91.8	100.9	30.0	-0.91	0.370
	morph*sex	412.7	257.7	661.8	1.60	0.110	726.9	1774.6	26.7	0.41	0.685
C_UV_	morph	0.041	0.002	664.8	17.42	< 0.001	15.01	14.99	27.9	1.00	0.325
	sex	-0.012	0.003	658.7	-4.62	< 0.001	9.96	15.72	28.9	0.63	0.531
	SVL	0.000	0.000	666.0	-1.01	0.312	0.84	1.05	28.7	0.80	0.428
	morph*sex	0.021	0.004	666.0	5.62	< 0.001	-27.19	19.17	256.0	-1.42	0.168
hue	morph	-4.55	2.49	665.8	-1.83	0.068	0.046	0.016	28.1	2.82	0.009
	sex	-9.74	2.84	659.9	-3.42	< 0.001	-0.006	0.017	29.0	-0.33	0.747
	SVL	0.62	0.22	665.1	2.85	0.005	-0.002	0.001	27.1	-1.28	0.212
	morph*sex	4.02	4.02	664.7	1.00	0.318	0.007	0.020	24.5	0.34	0.734

**Table 2:** Proportion of both white types in throats and bellies of *Podarcis muralis* males and females

	UV^+^white throat UV^+^white belly	UV^−^white throat UV^−^white belly	UV^−^white throat UV^+^white belly	UV^+^white throat UV^−^white belly
males	43.05%	47.53%	2.24%	7.17%
females	33.80%	26.85%	36.11%	3.24%

**Fig. 5:**
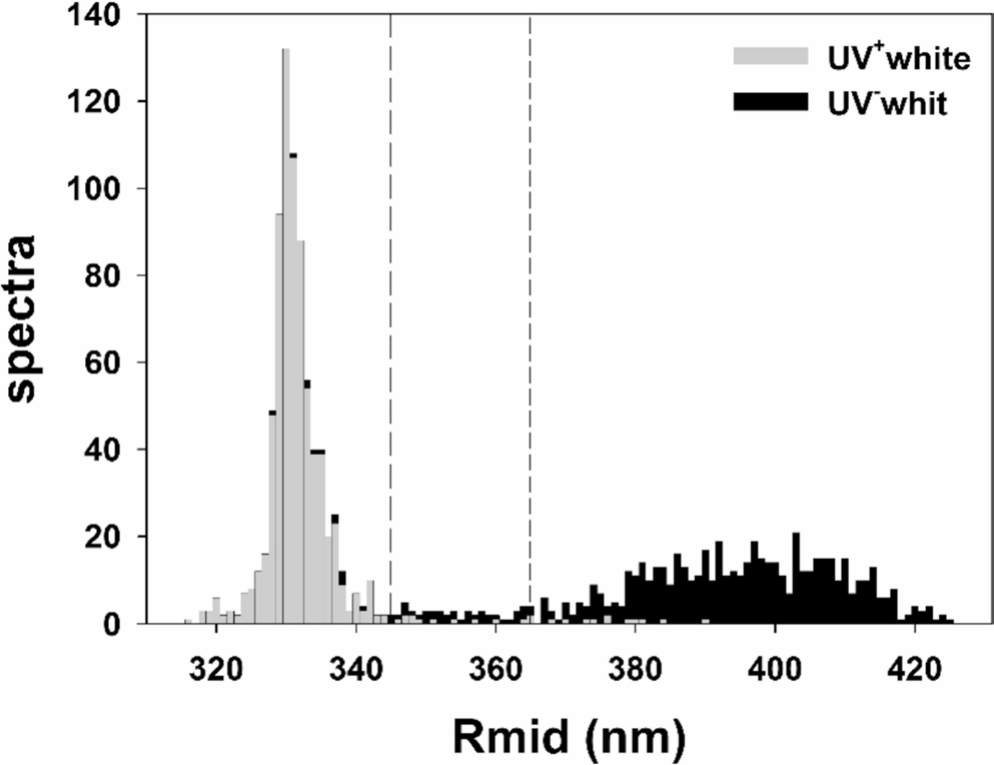
Distribution of R_mid_ values of the spectra of UV^−^white (black; *N* = 635) and UV^+^white (grey; *N* = 686) lizards. Note the bimodal distribution of the Rmid values. The sample combines spectra from throats and bellies of pure white animals and mosaic white- orange animals (spectra from the latter were taken from white patches)

Using R_mid_, 96.4% (*N* = 1321) of the spectra were classified below the UV^+^white threshold (i.e. 345 nm) or above the UV^−^white threshold (i.e. 365 nm), allowing their unambiguous assignment to one of the two categories. Figure 5 shows the distribution of R_mid_ values, which is clearly bimodal. According to the visual models, the two whites are chromatically discriminable by conspecifics (mean pairwise chromatic distance = 5.07 JND, bootstrapped 95% confidence intervals for mean distances between groups in colour space = 4.84 − 5.27; PERMANOVA: *F*_1,1361_ = 1368.7, *P* < 0.0001).

**Fig. 6:**
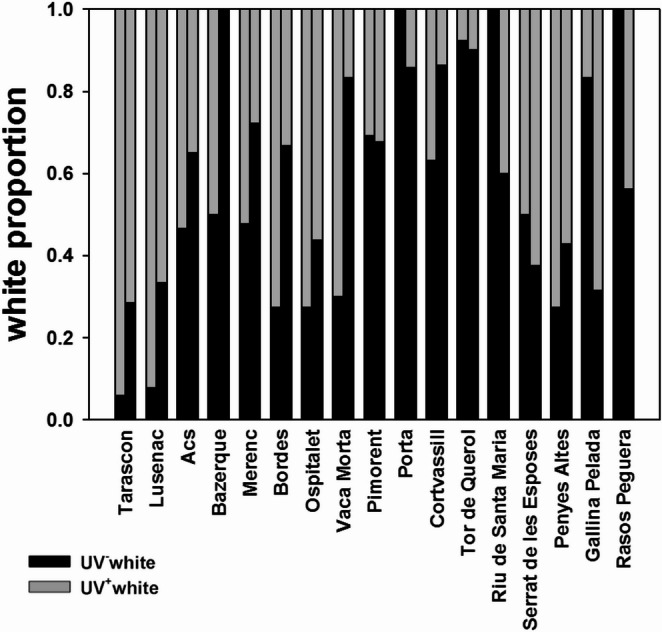
Stacked bar plot showing the proportion of UV^+^white and UV^−^white males and females of *Podarcis muralis* divided by sampling locality. For each locality, the first column represents data for males and the second for females. Sample sizes are detailed in Table S1

The proportion of the two whites in *P. muralis* shows marked geographic variation, with some localities holding almost no UV^+^white or UV^−^white lizards, such as Tor de Querol and Tarascon, respectively (Fig. 6).

**Fig. 7:**
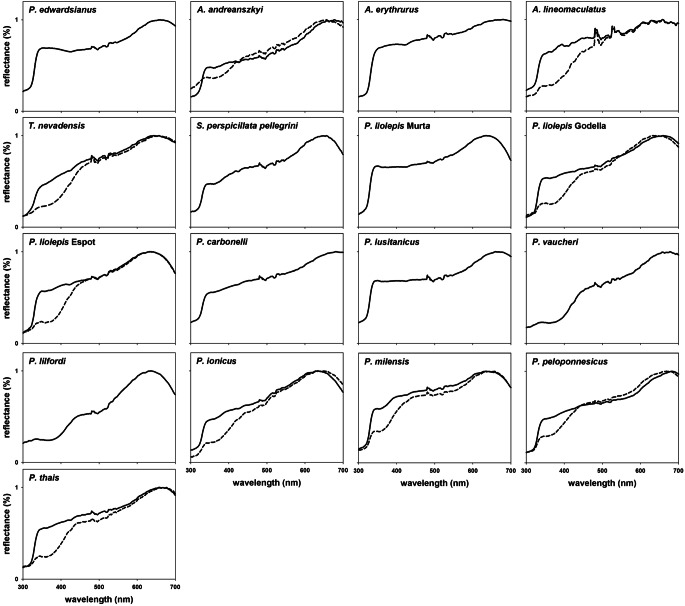
Normalized mean spectra from UV^+^white (continuous line) and UV^−^white (broken line) colorations of different lacertids. Each spectrum results of averaging all available spectra (pooling both sexes, throats and/or bellies) of a white type in a sample. Panels with a single spectrum correspond to species in which only one white is present. Sample sizes are detailed in Table S2

**Fig. 8:**
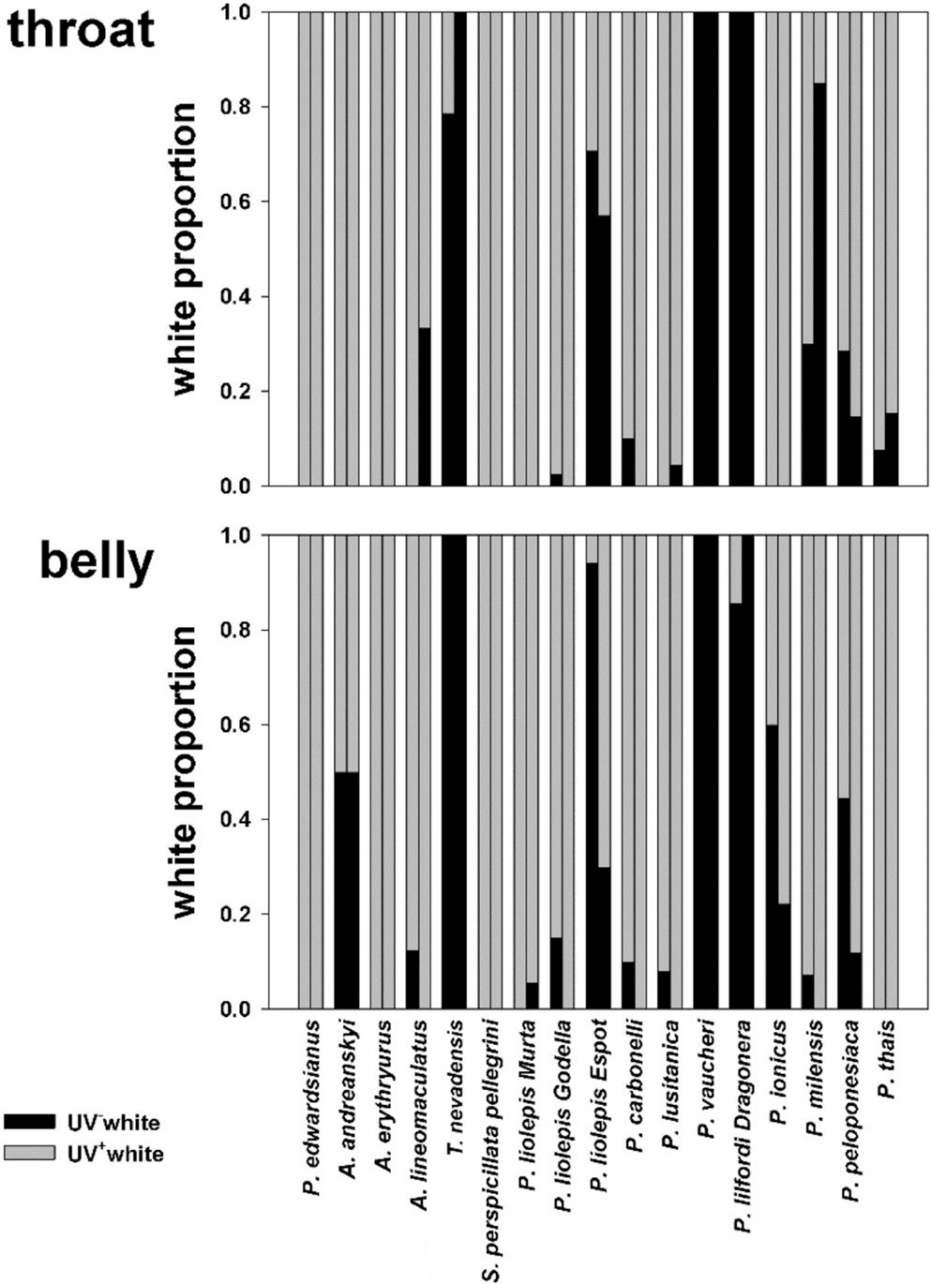
Stacked bar plot showing the proportion of UV^+^white and UV^−^white in the throat (up) and belly (down) of measured lacertid species. For each locality, the first column represents data for males and the second for females. Sample sizes are reported in Table S2

UV^−^white and UV^+^white are found in the ventral body surface of other lacertid species, often in lizards belonging to the same population (Fig. 7). The two types of white coloration are present in colour polymorphic species (e.g. *Podarcis liolepis*), as well as in species considered monomorphic (e.g. *P. milensis*; Fig. 8). Most species in our sample show UV^+^white, or both UV^+^- and UV^−^white. Two species (*P. lilfordi* and *P. vaucheri*) show only the UV^−^white phenotype. Our sample includes three different populations of *P. liolepis*, also showing great variation in morph composition (i.e. one monomorphic UV-white population and two white polymorphic populations, Fig. 7).

## Discussion

The results presented here demonstrate the existence of a cryptic polymorphism in the ventral coloration of adult *Podarcis muralis* involving two previously unrecognized alternative white morphs: one absorbing the UV wavelengths (UV^−^white) and the other reflecting it (UV^+^white). The UV^−^white morph corresponds to the phenotype identified as the white morph in most studies, whereas the UV^+^white had until now been described only in juveniles (Ábalos et al. 2025) and in the throat of some adult females (Pérez i de Lanuza et al. 2013a).Visual models suggest that lizards can discriminate these two whites as categorically distinct colours. Although a behavioural confirmation of colour discrimination is currently not available, the magnitude of the chromatic difference between both whites (the difference between spectral slopes is around 100 nm; Fig. 3) is similar to that observed between the white (i.e. UV^−^white) and yellow morphs (ca. 100 nm; see Fig. 2 in Pérez i de Lanuza and Font 2015), or between the yellow and orange morphs of this species (Pérez i de Lanuza and Font 2015), whose alternative ventral colours are discriminated by conspecifics (Pérez i de Lanuza et al. 2018a).

As both whites are simultaneously present in adult individuals from the same populations, we surmise that the two different whites correspond to two different colour morphs and therefore represent a case of cryptic polymorphism. Note that definitions of colour polymorphism only refer to the coexistence of distinct colour phenotypes within a breeding population (Ford 1945; Huxley 1955; Gray and McKinnon 2007; White and Kemp 2016; but see below). Whether or not the different morphs are associated with different life-history strategies or alternative reproductive tactics (as in the side-blotched lizard, *Uta stansburiana*; e.g. Sinervo and Lively 1996) is a different question. Previous attempts to unravel the functional significance of the colour polymorphism in *P. muralis* have produced inconclusive results and do not support the existence of alternative reproductive tactics linked to ventral colour variation in species (Ábalos et al. 2020). Our findings increase the complexity of the colour polymorphism described in *P. muralis* since animals visually assigned to the white category may in fact belong to two different morphs (UV^−^white and UV^+^white). The same applies to the white-orange mosaics, which can in fact be UV^−^white-orange or UV^+^white-orange. In contrast, no cryptic UV morphs were found among the yellow, orange, or yellow-orange lizards. This raises the number of potential colour morphs in *P. muralis* from the five usually recognized in the literature (e.g. Pérez i de Lanuza et al. 2013a) to seven: UV^−^white, UV^+^white, yellow, and orange pure morphs, and UV^−^white-orange, UV^+^white-orange, and yellow-orange mosaic morphs.

One reason why this cryptic polymorphism may have gone undetected until now is the existence of geographic variation in the frequencies of the white morphs. In our primary study area in the Pyrenees (i.e. central Cerdanya plateau), the prevalence of UV^+^white lizards is anecdotal (e.g. < 10% of white animals in the Tor de Querol population; Fig. 6). For this reason, when initially discovered, we considered this unusual colour an anomaly possibly related to some problem affecting colour development, similar to melanic or axanthic individuals (e.g. Ábalos et al. 2017). However, the data reported here show that the UV^+^white morph may be present in most white animals in some populations (up to 88% in Lusenac; Fig. 6), showing great variation across populations (see Table S1).

The coexistence of two white morphs in populations of *P. muralis* from the Pyrenees could have an impact on the study of the evolutionary causes and consequences of this polymorphism. Despite several decades of research on pigmentary colour polymorphism in this species, our understanding of the mechanisms allowing the coexistence of polymorphic coloration and driving its evolution is still incomplete. Cryptic morphs were a missing piece in this puzzle and could possibly contribute to a better understanding of this polymorphism. Future work should make the use of objective methods of colour analysis mandatory to properly assess the morph of white lizards. We should also revisit previous studies on *P. muralis* and other colour polymorphic lacertids to assess the extent to which this cryptic polymorphism affects conclusions regarding the evolution of the polymorphism.

Our findings also raise the question whether this cryptic polymorphism is genetically-determined (as is the case with the yellow and orange morphs; Andrade et al. 2019), this being a requirement to consider this chromatic variation part of a polymorphism and not a case of polyphenism, whereby some genotypes possess the ability to express varied phenotypes depending upon the environment. Identifying the cellular and subcellular mechanisms that produce UV^+^ white and UV^−^ white coloration is crucial to understanding its genetic bases and could also provide important insights into the evolutionary origins and functional significance of this polymorphism. Although the present results suggest a weak influence of body size and sex in the differential expression of both whites, this should be tested properly considering the impact of ontogeny and individual condition on colour expression. This is necessary to adequately determine the age (i.e. body size) at which the white morphs are fixed, which could differ from that observed for yellow and orange morphs (Pérez i de Lanuza et al. 2013a; Ábalos et al. 2025). Yellow and orange lizards do not change to a different colour morph once they reach adulthood (Pérez i de Lanuza et al. 2013a), but it is possible that the coexistence of two whites represents, at least in part, some type of plastic change. Testing these nonexclusive hypotheses requires longitudinal data from the same individuals, which unfortunately are not currently available.

Our preliminary survey suggests that the presence of both types of white is not exclusive of *P. muralis*, nor is it restricted to the genus *Podarcis*, although the available information is insufficient to discern whether this is an ancestral state or a trait that evolved multiple times convergently. While some lacertid species only show UV^+^white coloration (e.g. *Psammodromus edwarsianus*, *Acanthodactylus erythrurus*), white animals in other species are always UV^−^white (e.g. *Podarcis vaucheri*). However, the coexistence of UV^+^white and UV^−^white in the same species and population is also common, suggesting that these species should be considered colour polymorphic, even when they seem monomorphic to us (e.g. *Podarcis milensis*). This is of especial relevance for research focused on the coloration of lacertids, which are becoming a model system for the study of the macroevolution of colour polymorphisms (Stuart-Fox et al. 2021; de Solan et al. 2023).

Exploring other lizard clades for the presence of two white colorations may be also promising. In fact, two spectrally distinct whites (potentially similar to the UV^−^white and the UV^+^white of *P. muralis*) have also been reported in *Sceloporus* lizards (Hews and Martins 2013). In this case, females from some species present UV-absorbing white ventral coloration, whereas females from other species present UV-reflecting white coloration. Although this interspecific variation does not represent a polymorphism, we cannot discard the existence of intraspecific and intrapopulation cryptic colour variation in some *Sceloporus* species.

Judging from the vantage point of the present results, it is clear that previous studies of colour polymorphism in *P. muralis* (including our own) were afflicted by what Rivas and Burghardt aptly refer to as anthropomorphism by omission: the widespread tendency to judge other species’ perceptual worlds (*Umwelt*) based on our own (human) perception (Rivas and Burghardt 2002). This mistake has resulted in an oversimplification of the complexity of the colour polymorphism in this species and possibly also in other polymorphic taxa. By ignoring the point of view of the species of interest we can incur in misinterpretations in the description and assessment of the evolutionary processes underlying the emergence and maintenance of polymorphisms. Thus, we strongly encourage researchers to explore the colours under study using objective measures such as reflectance spectrophotometry and full-spectrum photography (e.g. Stevens et al. 2007), even when working with common and well-known species. To avoid anthropomorphism by omission we must take into account the sensory world of the species we study, a widely accepted maxim that, unfortunately, is not always heeded (Partan and Marler 2002; Bueno-Guerra and Amici 2018; Brebner et al. 2024).

## Supplementary Information

Below is the link to the electronic supplementary material.

Supplementary Material 1 (DOCX 736 KB)

## Data Availability

All data supporting the findings of this study are available within the paper and its Supplementary Information.
