# Supplementary material for: More than meets the (human) eye: Cryptic chromatic diversity in a colour polymorphic lizard: Cryptic polymorphism in lacertid lizards
Source: Naturwissenschaften. 2026 May 7;113(3):58. doi: 10.1007/s00114-026-02106-2 (PMC13152896; doi:10.1007/s00114-026-02106-2)
Supplement: Supplementary file 1 — Supplementary Material 1 (DOCX 736 KB) [file 114_2026_2106_MOESM1_ESM.docx]

**
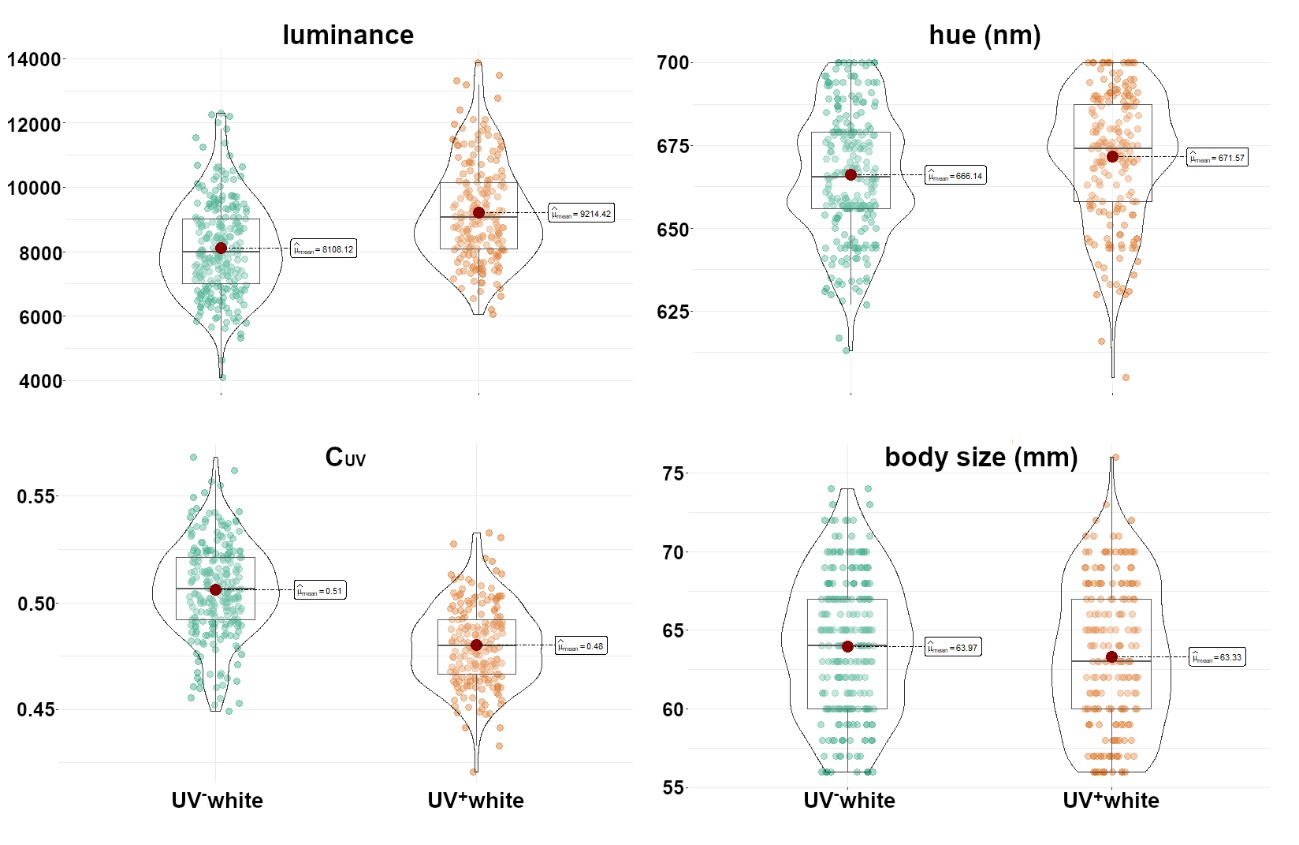
**

**Figure S1.** Violin plots comparing luminance, hue, C_UV_, and body size of UV^-^white and UV^+^white throats in *Podarcis muralis* lizards. Sample size of UV^-^white lizards = 245 (110 males + 135 females); sample size of UV^+^white lizards = 195 (115 males + 80 females).


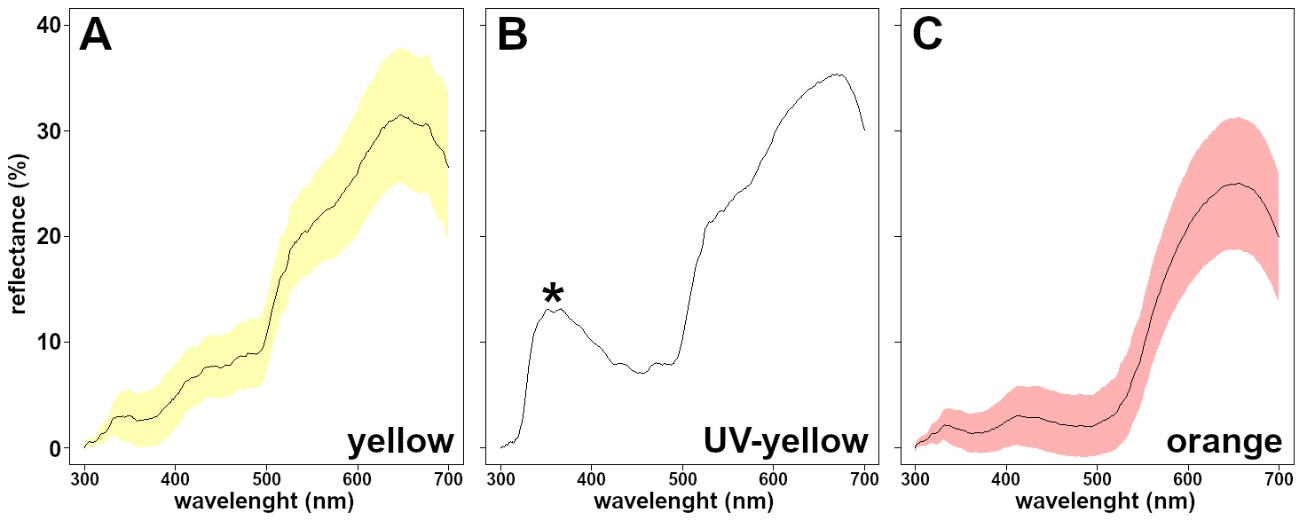


**Figure S2.** Reflectance spectra from throats other than white in *Podarcis muralis*. A) Yellow animal (*N* = 99); B) the only individual out of a total of 100 yellow animals with a secondary UV peak highlighted by an asterisk; C) orange animals (*N* = 325). Grey lines represent de median and yellow and reddish bands represent standard deviation.


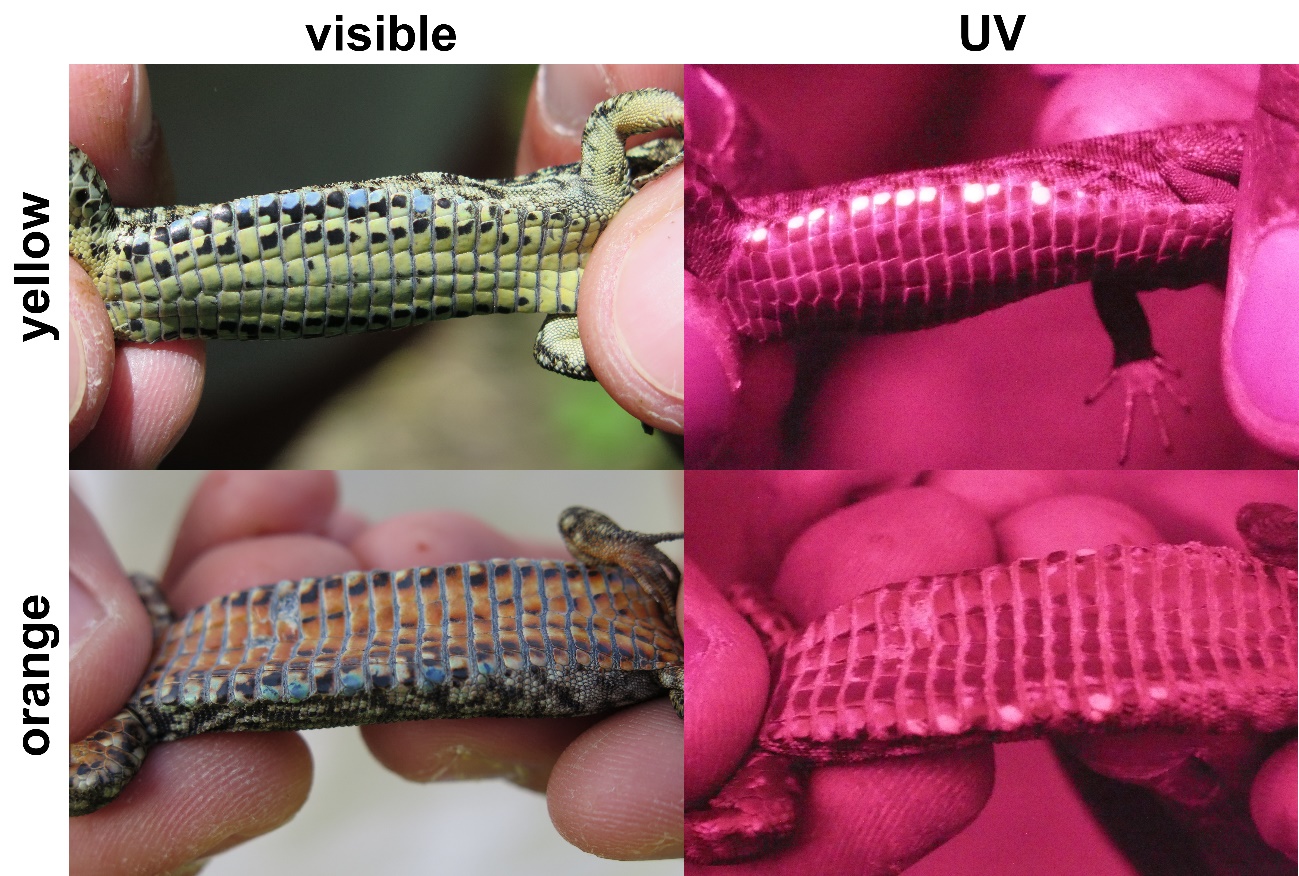


**Figure S3.** Representative pictures of male *Podarcis muralis* showing the yellow phenotype (top) and the orange phenotype (bottom), in the visible spectrum (i.e. 400-700 nm; left) and in the near UV spectrum. The lizards’ head is to the right in all the pictures. Note that both bellies look dark because they absorb UV (compare their coloration to that of the highly UV-reflecting ventrolateral blue patches). Pictures in the human-visible range were obtained with a standard digital camera (Canon PowerShot G16). UV pictures were obtained with a digital camera (Olympus PEN Mini) with the standard internal hot mirror filter replaced by a Spectrosil 2000 fused silica filter, fitted with a UV-transmitting lens (Noflexar Novoflex 1:3,5/35mm) and a Baader U-filter with peak transmission at 350 nm (ca. 80%) and a bandwidth of 60 nm (between 320 and 380 nm). UV photographs were taken outdoors in the shade using natural illumination.

**Table S1.** *Podarcis muralis* classified by locality, sex and white type. Lizards were classified based on throat coloration, regardless of their belly coloration, which is UV^+^white in almost of the females. UV^-^white = UV^-^W; UV^+^white = UV^+^W; UV^-^white-orange mosaic = UV^-^WO; UV^+^white-orange mosaic = UV^+^WO. Other morphs include orange, yellow and orange-yellow lizards. The apparent mismatch between these sample sizes and those reported in Table 1 results from females showing other colours contributing with their UV^+^white bellies to the overall analyses.

|  | **white** | | | | **white-orange mosaics** | | | | **other morphs** | |  |  |
| --- | --- | --- | --- | --- | --- | --- | --- | --- | --- | --- | --- | --- |
|  | **males** | | **females** | | **males** | | **females** | | **males** | **females** |  |  |
| **locality** | **UV^-^W** | **UV^+^W** | **UV^-^W** | **UV^+^W** | **UV^-^WO** | **UV^+^WO** | **UV^-^WO** | **UV^+^WO** |  |  | **longitude** | **latitude** |
| Tarascon | 1 | 16 | 2 | 5 | 1 | 0 | 2 | 10 | 8 | 7 | 1.6106 | 42.8399 |
| Lusenac | 1 | 12 | 1 | 2 | 1 | 0 | 2 | 1 | 17 | 16 | 1.7628 | 42.7619 |
| Acs | 7 | 8 | 13 | 7 | 1 | 0 | 7 | 1 | 16 | 11 | 1.8322 | 42.7245 |
| Bazerque | 8 | 8 | 6 | 0 | 0 | 0 | 6 | 2 | 21 | 15 | 1.8276 | 42.6977 |
| Merenç | 10 | 9 | 13 | 5 | 0 | 1 | 10 | 2 | 19 | 8 | 1.8376 | 42.6571 |
| Bordes | 3 | 8 | 2 | 1 | 1 | 0 | 3 | 1 | 26 | 8 | 1.8253 | 42.6310 |
| Ospitalet | 6 | 16 | 7 | 8 | 4 | 4 | 6 | 2 | 19 | 4 | 1.7996 | 42.5900 |
| Vaca Morta | 3 | 7 | 5 | 1 | 0 | 2 | 4 | 3 | 20 | 10 | 1.7723 | 42.5664 |
| Pimorent | 8 | 4 | 21 | 10 | 2 | 2 | 6 | 5 | 7 | 18 | 1.8138 | 42.5606 |
| Porta | 4 | 0 | 12 | 2 | 1 | 2 | 7 | 6 | 22 | 13 | 1.8240 | 42.5261 |
| Cortvassill | 12 | 7 | 19 | 3 | 8 | 3 | 7 | 1 | 17 | 10 | 1.8505 | 42.4926 |
| Tor de Querol | 11 | 0 | 9 | 1 | 1 | 0 | 3 | 2 | 28 | 18 | 1.8889 | 42.4632 |
| Riu de Santa Maria | 5 | 0 | 2 | 2 | 6 | 3 | 3 | 2 | 25 | 10 | 1.7876 | 42.3674 |
| Serrat de les Esposes | 10 | 10 | 6 | 10 | 3 | 0 | 5 | 3 | 10 | 5 | 1.8255 | 42.3230 |
| Penyes Altes | 3 | 8 | 3 | 4 | 0 | 1 | 1 | 1 | 6 | 1 | 1.8390 | 42.3049 |
| Gallina Pelada | 10 | 2 | 6 | 12 | 1 | 0 | 0 | 7 | 10 | 7 | 1.7518 | 42.1997 |
| Rasos de Peguera | 9 | 0 | 8 | 7 | 2 | 0 | 3 | 0 | 12 | 9 | 1.7645 | 42.1430 |
| **Total** | **111** | **115** | **135** | **80** | **32** | **18** | **75** | **49** | **283** | **170** |  |  |

**Table S2.** Lacertid species examined with sample sizes classified by sex (m = males, f = females), body region, and white type.

| **species** | **sex** | **throat** | | **belly** | | **longitude** | **latitude** |
| --- | --- | --- | --- | --- | --- | --- | --- |
|  |  | **UV^-^white** | **UV^+^white** | **UV^-^white** | **UV^+^white** |  |  |
| *Psammodromus edwardsianus* | m | 0 | 26 | 0 | 24 | -0.3132 | 39.3410 |
|  | f | 0 | 11 | 0 | 11 |  |  |
| *Atlantolacerta andreanskyi* | m | 0 | 6 | 3 | 3 | -7.8565 | 31.1968 |
|  | f | 0 | 5 | 2 | 2 |  |  |
| *Acanthodactylus erythryurus* | m | 0 | 27 | 0 | 27 | -0.3132 | 39.3410 |
|  | f | 0 | 11 | 0 | 11 |  |  |
| *Acanthodactylus lineomaculatus* | m | 0 | 7 | 1 | 7 | -6.3002 | 34.8647 |
|  | f | 1 | 2 | 0 | 3 |  |  |
| *Timon lepidus* | m | 11 | 3 | 18 | 0 | -0.1143 | 38.9048 |
|  | f | 11 | 0 | 11 | 0 |  |  |
| *Scelarcis perspicillata pellegrini* | m | 0 | 10 | 0 | 10 | -4.0726 | 34.1042 |
|  | f | 0 | 2 | 0 | 2 |  |  |
| *Podarcis liolepis* (la Murta) | m | 0 | 28 | 0 | 28 | -0.3729 | 39.1329 |
|  | f | 0 | 16 | 1 | 17 |  |  |
| *Podarcis liolepis* (Godella) | m | 1 | 38 | 6 | 34 | -0.4197 | 39.5185 |
|  | f | 0 | 22 | 0 | 29 |  |  |
| *Podarcis liolepis* (Espot) | m | 17 | 7 | 16 | 1 | 1.0858 | 42.5777 |
|  | f | 4 | 3 | 6 | 14 |  |  |
| *Podarcis carbonelli* | m | 1 | 9 | 1 | 9 | -8.7143 | 40.7567 |
|  | f | 0 | 7 | 0 | 7 |  |  |
| *Podarcis lusitanica* | m | 0 | 25 | 2 | 23 | -8.8613 | 41.8486 |
|  | f | 1 | 21 | 0 | 22 |  |  |
| *Podarcis vaucheri* | m | 14 | 0 | 14 | 0 | -7.8565 | 31.1968 |
|  | f | 10 | 0 | 12 | 0 |  |  |
| *Podarcis lilfordi gigliolii* | m | 13 | 0 | 6 | 1 | 2.3279 | 39.5878 |
|  | f | 12 | 0 | 10 | 0 |  |  |
| *Podarcis ionicus* | m | 0 | 20 | 3 | 2 | 22.3257 | 37.9463 |
|  | f | 0 | 6 | 6 | 21 |  |  |
| *Podarcis milensis* | m | 6 | 14 | 2 | 26 | 24.4567 | 36.7270 |
|  | f | 17 | 3 | 0 | 22 |  |  |
| *Podarcis peloponesiaca* | m | 2 | 5 | 4 | 5 | 22.3257 | 37.9463 |
|  | f | 5 | 29 | 5 | 37 |  |  |
| *Podarcis thais* | m | 2 | 24 | 0 | 24 | 37.8569 | 22.4553 |
|  | f | 4 | 22 | 0 | 25 |  |  |
